# Sunlight-Operated TiO_2_-Based Photocatalysts

**DOI:** 10.3390/molecules25174008

**Published:** 2020-09-02

**Authors:** Irene Barba-Nieto, Uriel Caudillo-Flores, Marcos Fernández-García, Anna Kubacka

**Affiliations:** 1 Instituto de Catálisis y Petroleoquímica (CSIC), C/Marie Curie 2, Cantoblanco, 28049 Madrid, Spain; irene.barba.n@gmail.com (I.B.-N.); ucaudillo@gmail.com (U.C.-F.); 2 Centro de Nanociencias y Nanotecnología, Universidad Nacional Autónoma de México, Ensenada 22800, Mexico

**Keywords:** photocatalysts, titanium oxide, anatase, doping, composites, sunlight

## Abstract

Photo-catalysis is a research field with broad applications in terms of potential technological applications related to energy production and managing, environmental protection, and chemical synthesis fields. A global goal, common to all of these fields, is to generate photo-catalytic materials able to use a renewable energy source such as the sun. As most active photocatalysts such as titanium oxides are essentially UV absorbers, they need to be upgraded in order to achieve the fruitful use of the whole solar spectrum, from UV to infrared wavelengths. A lot of different strategies have been pursued to reach this goal. Here, we selected representative examples of the most successful ones. We mainly highlighted doping and composite systems as those with higher potential in this quest. For each of these two approaches, we highlight the different possibilities explored in the literature. For doping of the main photocatalysts, we consider the use of metal and non-metals oriented to modify the band gap energy as well as to create specific localized electronic states. We also described selected cases of using up-conversion doping cations. For composite systems, we described the use of binary and ternary systems. In addition to a main photo-catalyst, these systems contain low band gap, up-conversion or plasmonic semiconductors, plasmonic and non-plasmonic metals and polymers.

## 1. Introduction

Heterogeneous photocatalysis uses semiconductors to generate charge carrier species from the absorption of light. These charge carriers can migrate to the surface of the photo-catalysts and transform chemical molecules in the context of chemical reactions concerning elimination of pollutants, generation of fuels or added value chemicals, microorganism inactivation, and others. All these chemical (and biological) processes compete with the recombination of charge, and the balance between these two types of processes controls the fate of charge carrier species and primarily determine photo-activity [1,2,3].

The most successful semiconductor used corresponds to titania. Titania can present at least three stable polymorphs: anatase, rutile and brookite. The first is considered the most active and has broad utility due to its good performance in all reaction tested, its wide availability, reduced cost and relatively limited effects in human health. Other interesting oxides correspond to zinc, tungsten, cerium, bismuth or most complex ones like perovskites, tungstates, bismutates, etc. Additional non-oxide semiconductors broadly utilized correspond to carbon nitride, graphene or polyoxometalates [1,4,5,6,7,8,9,10,11]. All these systems share a main problem; they all can utilize a single range of sunlight wavelengths. Specifically, most of them can use UV or visible light and a few can profit from near infrared, however none of them can effectively (for example, visible light active bismutates cannot perform as titania under UV and vice versa) use two of them, nor the complete range of wavelengths going from UV to infrared ranges.

The achievement of this goal, i.e., the fruitful use of solar light in photocatalysis, is a main subject of the research are and it is here analyzed. Of course, the literature work is immense, and we will select particularly relevant or recent studies. Moreover, in relation with the full use of the electromagnetic spectrum of sunlight a critical point is dismissed sometimes. Recent photocatalytic materials attempt to concentrate in achieving visible or near infrared active materials as the corresponding light ranges correspond roughly to 40–45 and 50–55% of the solar light intensity at sea level. However, when tested simultaneously, it is typically shown that being UV active is critical if measured by the true quantum efficiency. A clear comparison has been presented showing that this is the case, for example, for hydrogen photoproduction. In this case, the quantum efficiency values under a 1.5AM solar-type illumination can have a significant contribution from the UV part in several highly-active TiO_2_-based composite photocatalysts [12], in spite of the fact that the UV part of the sunlight intensity is only around ca. 4%. This would indicate that using the full range of wavelengths efficiently requires the development of visible or near infrared efficient materials but without dismissing UV activity. This short discussion points out that titania can be the base of most active materials. In fact, a survey of literature summarized in review articles indicates that this is the general case in photocatalysis [1,2,3].

Titania alone, and particularly the anatase phase, can have UV and visible activity if we obtain a nanostructured material. Nanostructured anatase typically shows a band gap energy of ca. 3.0–3.2 eV, while rutile showed up a slightly lower band gap energy. A decrease of the band gap energy would allow a profit for sunlight [1]. Size effects in titania-based materials band gap energy are complex, with a Bohr radius (defining the point below which strong quantum confinements effects are present) at ca. 4 nm [1,13]. The absorption of visible light for semiconductor single phase in anatase, rutile and any other (UV-absorbing) semiconductor is typically connected with the adequate engineering of a defect structure. The latter has been exploited and, probably, the most successful approach corresponds to black titania. This is essentially a defective, core-shell titania, mostly (although not exclusively) anatase-type structure, with several types of defects (hydrogen inclusion, anion vacancies, reduced Ti species, atom displacements from pure phase positions, etc.) concentrated at near surface sites. This allows us to significantly decrease the band gap energy up to ca. 1.5–2.5 eV and thus enables a significant decrease of band gap energy [14]. This procedure has been extended to other oxides such as ZnO or ZrO_2_ [15,16]. Still, a relatively inefficient use of the visible region, the limited stability of the defects and thus the usefulness of such type of “black” system are open questions, particularly in continuous flow operation or in oxygen-rich atmospheres during long-term stream periods. As consequence, black semiconductors cannot be used with the generality (all types of reactions above mentioned) as other potential technologies [17]. Such potential technologies relay in two main physico-chemical effects. The first is the surface and/or bulk doping of the high active photocatalysts phases and, to a large degree, of the anatase structure. The second is the generation of composite structures having anatase as one of the components. The first cases attempt to modify in several ways the electronic structure of the anatase (or any other single phase semiconductor) as well as create new surface active centers. The second objective can be shared by composite systems in which, additionally, the contact between phases as well as the optical properties of the additional phases can play key roles for photocatalysis. Here, we will review these two main approaches, attempting to discuss a selection of (the immense number of) relevant results published, here selected with the aim of stablishing appropriate activity-structure links which can help to rationalize the research field and would allow the future progress of the sunlight-operated photocatalysis field. Of course, in some cases, doping and composite systems are used together and here they will be classified considering the most relevant influence on photocatalytic properties.

## 2. Doped Photocatalysts

Doping is probably one of the most successful methods used to improve photoactivity of reference oxides such as titanium or zinc oxide. An early work tested the catalytic performance of nanostructured (ca. 5 nm) anatase at 0.1–0.5 at. % doping levels for 24 cations. Systems containing Fe^3+^, Mo^5+^, Ru^3+^, Os^3+^, Re^5+^, V^4+^, and Rh^3+^ showed increasing activity in hydrogen production with respect to the titania reference systems. However, they exhibited a complex dependence on doping ion concentration, state in the lattice and/or surface/bulk, d-electron configuration, light intensity and others physico-chemical variables [18]. Similarly, the doping of the nanostructured TiO_2_ anatase phase (5–13 nm interval) was achieved with nine cations, but in this case in a broad range of doping concentrations from ca. 1 to 25 mol% on cationic basis [19]. In general, a main objective is the modification of the band gap energy of the parent photocatalysts by altering conduction and/or valence band edge energies. This will lead to the desired decrease of band gap energy allowing simultaneous UV and visible light absorption for wavelengths typically in the range from 300 to ca. 550 nm, improving the profit of the solar light. Alternatively, the presence of dopant-associated gap states can produce transitions to or from the corresponding band of the solid, leading to visible light absorption.

**Figure 1:**
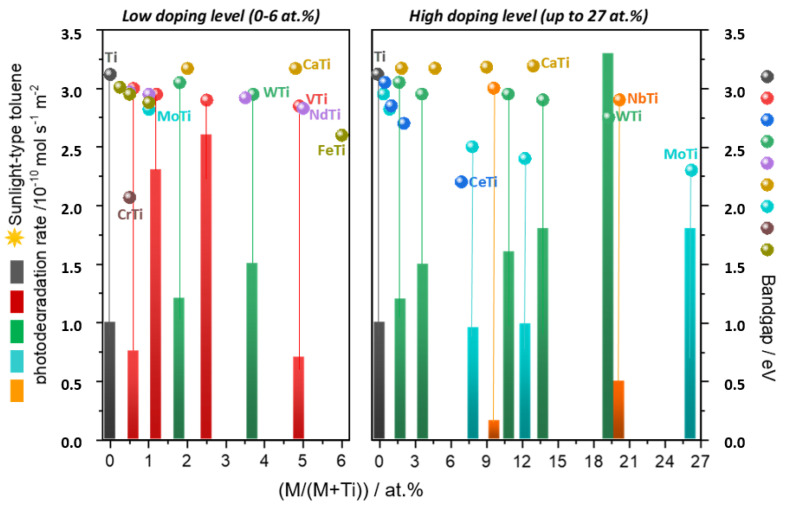
Band gap energy and photoactivity under sunlight-type irradiation vs. doping level for metal-doped TiO_2_ systems.

Cations tested are typical donors of charge (V^5+^, Mn^n+^), acceptors (Cr^3+^, Ni^2+^), and cations with both properties (Fe^2+/3+^) or with intrinsic photocatalytic activity (Nb^5+^, Ce^4+^, Mo^6+^ or W^6+^). Doping using V^4+^, Mo^6+^, and W^6+^ as cations was found to enhance photo-activity in toluene photo-degradation. For vanadium and tungsten, doping cations replace Ti ions at the anatase structure rendering substitutionally disordered mixed oxides. Maximum doping concentrations trigger optimum activity through a minimum disturbance of the long-range structure of anatase and defined local ordering favoring hetero M-O-Ti (vs. M-O-M) bonds. In the case of W, there were Ti vacancies as charge neutrality defects [20,21]. Figure 1 reflects the activity enhancement observed for materials having these structural characteristics. In other cases we can see, for example, that: Cr (dominant Cr^3+^ oxidation state with presence of Cr^6+^ typically growing with the Cr content) is only marginally included in the anatase lattice, Ca^2+^, V^4+^ (changing oxide state at surface to V^5+^), Nd^3+^ present a solubility limit of ca. 4–5 mol% and Mo (Mo^5+^ and Mo^6+^), Nb^5+^ and W^6+^ display up to 20–25 mol% solubility limit(s) [19,20,21,22]. As a general rule, optimum photoactivity under sunlight illumination can be connected with the limited structural disturbance of the anatase structure but with an effect in band gap energy. The structural stability of anatase limits the (potential) loss of efficiency using UV photons while the diminishing band gap energy allows the use of visible light for excitation of the semiconductor. Thus, combination of correct structural and electronic parameters within purely substitutionally disordered mixed oxides appears as an efficient way to profit from solar light.

Contrarily to the above-mentioned cases, Cu^2+^ [23], Zn^2+^ [24] or Zr^4+^ [25] doping of the anatase appear to mainly generate gap localized (electronic) states. In these cases, Cu doping was tested in toluene photo-degradation, with activity presenting a maximum at low loading (below 0.5 mol%) and strong deterioration for higher loadings, in presence of Cu-Cu interaction(s) [23]. In the case of Zn, the photo-degradation of phenol was essayed, with a sample having a 5 mol% of Zn displaying maximum activity. Above this concentration, presence of the cations in the samples leads to Zn accumulation at the surface, deteriorating the surface area and increasing the quantity of recombination centers [24]. Zr substitution of Ti cations favors the presence of localized states, likely near the valence band, increasing both UV and visible light absorption capability, decreasing recombination and favoring photoactivity [25].

Work carried to dope the ZnO oxide (wurtzite phase in most cases) is also of interest in photocatalysis. The physico-chemical situation is completely parallel to the titania case. Successful incorporation and concomitant increase of photoactivity have been observed, for example, using Al, Fe, Cu, Nd, and Ce [26,27,28,29]. Doping of other photocatalytic semiconductors is less studied although there are many examples such as the doping of bismuth oxide [30], thin oxide [31], cadmium sulfide [32] or of more complex formulations such as LaFeO_3_ [33].

Interestingly, some works are also devoted to use near-infrared wavelengths in addition to the UV and/or visible ones. Tm, Gd, Er and Ho (and combinations with other lanthanides such as Er/Yb) are cations specifically designed to increase activity under visible and near-IR illumination due to an up-conversion process that render UV or high-energy visible photons (all above ca. 380 nm) utilized by the anatase network. Specific cations (Like Pr and Nd) can also provide visible to UV up-conversion. All these cations are usually utilized in relatively low concentrations (typically between 0.5 mol% and 3 mol%) and pure anatase and anatase plus rutile composite powders are commonly doped with these lanthanides [34,35]. Other semiconductors extensively explored in the field of up-conversion though doping are, for example, BiVO_4_ [36] or Bi_2_WO_4_ [37] or CaF_2_ [38].

**Figure 2:**
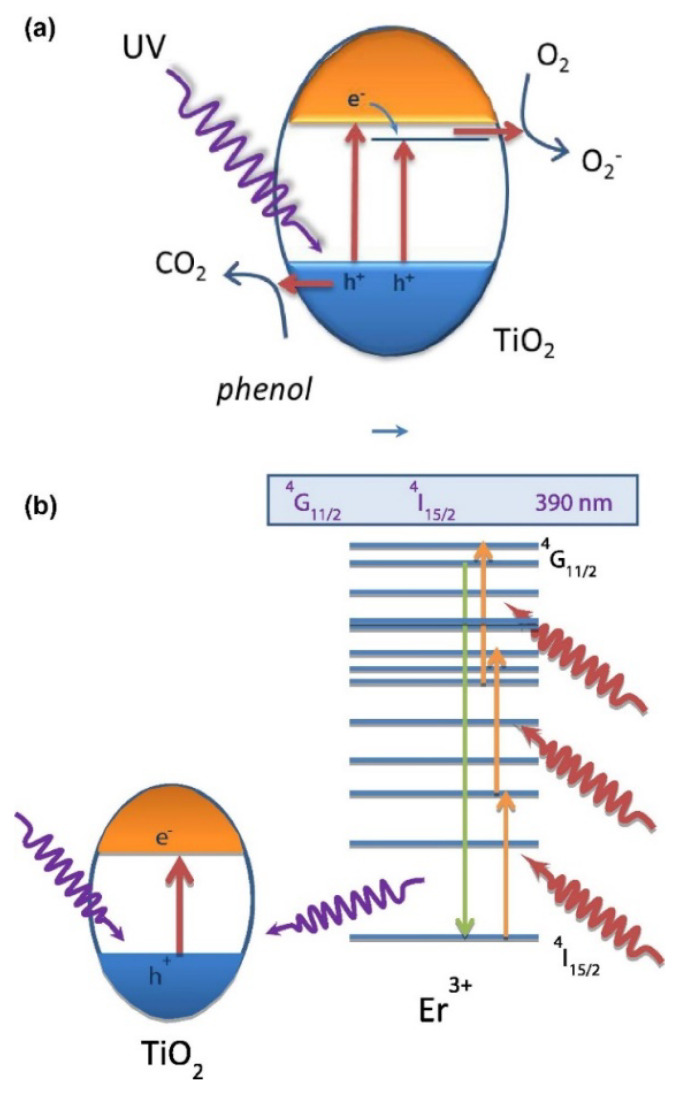

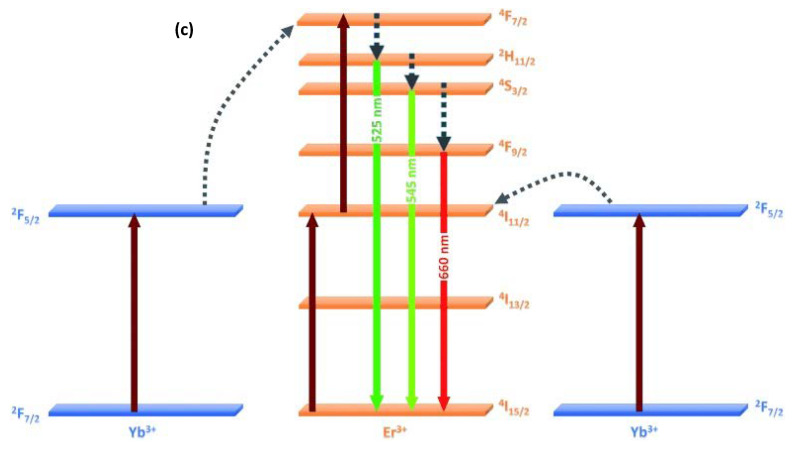
Proposed electronic mechanism for Er^3+^ doping of TiO_2_ occurring under UV excitation (**a**) and near-infrared (NIR) excitation (**b**). Reprinted with permission from [38]: Obregón, S.; et al. *J. Catal.* **2013**, *299*, 298–306. Copyright © 2020 Elsevier. Schematic of the upconversion mechanism in Er^3+^/Yb^3+^ co-doped materials (**c**). Reprinted with permission Naccache, R.; et al. *ChemSusChem*, **2013**, *6*, 1308–1311. Copyright © 2020 Wiley [34].

The Er case is the prototypical cation used for titania-based materials in the field of up-conversion. Erbium-doped TiO_2_ materials showed good photoactivity for the liquid-phase degradation of phenol and methylene blue and the gas phase of toluene. The characterization results indicated that the presence of Er^3+^ induces a progressive anatase cell expansion due to its incorporation in the TiO_2_ lattice. The best photocatalytic performance was attained for the samples with 2 at. % of Er^3+^ irrespective of the chemical degradation reaction essayed. Er presence increases simultaneously UV and NIR activity, as schematically shown in Figure 2 (panels a and b). The first is based on the positive effect of Er doping ions acting as electron scavenger and decreasing charge recombination. The up-conversion luminescence process of Er^3+^ also allowed us to profit in terms of the NIR range of the lamp and transferring energy in the UV range to the TiO_2_. This indicates that Er^3+^ plays a significant role in the overall photocatalytic mechanism [39]. Er is commonly essayed in presence of Yb. The benefit is graphically explained in Figure 2c. Er and Yb co-doping titania (2 at. % Er and 0, 10, 15, or 20 at. % Yb), obtained by a modified hydrothermal process, was essayed on photocatalytic degradation of phenol and Rose Bengal dyes under simulated solar radiation and using LED illumination of specific frequencies in the ultraviolet, green, red, and infrared regions. The 2% Er 10%Yb co-doped TiO_2_ catalyst exhibited the best phenol and Rose Bengal photo-degradation under UV illumination. However, using green, red, and infrared illumination no significant activity was observed [40]. The Er, Yb, and Er-Yb titania powders were tested under visible light excitation showing that catalysts can work with illumination wavelengths up to ca. 475 nm and that Yb at 1 at. % rendered the best activity [41]. Thus, no obvious improvement over Er-doped materials appears in the case of Er-Yb co-doped titania photocatalysts. Also a Er-Sm co-doped anatase-rutile composite (mixed anatase-rutile with dominant presence of the first) catalyst have been analyzed in the literature [42]. The catalyst having a 3/0.6 mol% of Sm/Er maximizes activity for degradation of the acid blue 113 dye working at pH 2. Sm presence triggers the decrease of anatase band gap to reach a value of 2.8 eV, opening the fruitful use of visible photons. At the same time, Er is responsible for an up-conversion process which generates UV from IR photons, providing energy to the anatase powder.

Additional research towards the full use of the solar spectrum explored the co-doping of titania materials having Er and other non-luminescent cations. Er was used together with W to dope anatase nanostructured materials. In this case, nanostructured (ca. 10 nm) anatase powders were synthesized in presence of Er (2 mol%) and W (ca. 15 mol%) [43]. The structural investigation demonstrates the substitutional replacement of Ti by both cations with presence of characteristic Er-O-W contacts in the co-doped system. The system showed outstanding performance against Gram-positive and Gram-negative bacteria. A joint use of EPR and photoluminescence rendered information about charge carrier species generated after illumination. Activity showed an acute dependence of the excitation wavelength due to a different physical origin. Below ca. 550 nm (so, in the UV or initial visible region) a strong decrease of recombination and generation of surface local entities promoting the formation of hydroxyl radicals were directly ascribed to the substitutional replacement of Ti ions of the anatase structure. For most of the visible (above 550 nm) as well as for infrared wavelengths up to ca. 1000 nm, additional surface sites linked with W-Er surface species were responsible for generation of hot electron species. The two types of photo-related mechanisms inactivate the microorganisms and render a material able to work efficiently in the whole UV-visible-near IR spectral range [43]. Another case study corresponds to Er-Ce co-doping of titania. In this case, the Er:Ce atomic ratio (Er 0.5 mol% and Ce 0.1, 0.2 and 0.3 mol%) was varied. The materials were employed for methylene orange photo-degradation and photocatalytic disinfection (*S. aureus* and *E. coli*) tests. In all photocatalytic tests, the sample Er/Ce = 0.5/0.2, calcined at 800 °C, exhibited the highest photocatalytic performances. The existence of Er ions is thought to successfully turn the near-infrared radiation into visible region, which is easier to be absorbed by the co-doped TiO_2_ material. Meanwhile, the addition of Ce ions can effectively extend spectral response range and inhibit the recombination of electrons and holes, enhancing the photocatalytic disinfection activity of co-doped TiO_2_ [44].

The utilization of visible and, critical considering the goal of this contribution, sunlight photons has been also deeply investigated using non-metal (also called anion) doping. An interesting approach attempted to control the formation of the Ti-N bond in the catalyst using N-containing-type ligands (2-methoxyethylamine, tetramethylethylenediamine and 1,2-phenylenediamine) in the initial Ti precursor subjected to hydrolysis in a microemulsion medium and posterior calcination [45,46]. The method renders anatase materials with (size in the 8–14 nm), tested in the photo-degradation of methylcyclohexane. Light absorption into the visible region was achieved in all cases, but activity was maximized using the tetramethyl-ethylenediamine ligand. Activity under solar illumination was associated with N incorporation to anatase lattice and presence of charge neutrality vacancies. A multi-edge X-ray absorption study of the catalysts provided insights into the charge neutrality process, demonstrating the existence of cationic vacancies as well as strongly disturbed middle range order with absence of typical Ti (III) states. Optimum activity was found to be associated with an optimum N/Ti ratio of ca. 0.1. Higher N content leads to interacting defects affecting negatively activity by an increase of the charge recombination [47]. A combination of microemulsion and hydrothermal synthesis procedures utilized different (organic/inorganic molecule) sources in the aqueous phase to dope titanium oxide with nitrogen. Materials were tested for Rhodamine B (RhB) dye and 2,4-dichlorophenol elimination. Maximum activity was obtained with a trimethylamine precursor used as nitrogen source. Band gap modification and charge recombination decrease were detected as a result of nitrogen incorporation. Both render positive activity effects for a (synthesis) N/Ti ratio of 2 [48]. More simple preparation procedures consist of thermal treatment of titania oxo-hydroxide precursor(s) in ammonia, hydrazine, nitrous oxide and/or other nitrogen-containing gases or liquids [49]. Similarly, other oxides such as ZnO, perovskites and many others have been examined in the context of nitrogen doping [50]. In general, and as summarized in references [1,49], nitrogen doping is a rather complex phenomenon from a structural and electronic point of view. Several chemical species (N at anion position, NO, NH_x_, CN at interstitial and surface positions, and others) are present in the solid irrespective of the preparation procedure leading to a variety of situations. However, it appears that, essentially, such N-containing species create different localized defects states [1,45,46,47,48,49,50]. Of course, this depends on the loading, as it grows localized states located near the valence and conduction bands of parent structure can coalesce with such bands, changing from localized to delocalized structure. However, it appears that in non-covalent oxides such as titanium or zinc oxides a high level of nitrogen, above ca. 2–3 at. %, is required for such coalescence and thus mostly localized states are obtained in reported publications [1,49,50]. Figure 2 depicts a summary of the (main) chemical species and corresponding (localized) electronic effects affecting activity. Simultaneous use of UV and visible photons is ascribed to a combination of effects related to the new states near the conduction band (absorption of visible light photons) and, at specific N/Ti ratios, a decrease of charge recombination, obtained irrespective of the illumination wavelength. Other species may also contribute to enhance the photo-activity but the corresponding structure-activity links are yet to be firmly established. The combination of N and cations such as W was essayed, and the study of the corresponding preparation procedure and chemical species present in the solid thoroughly detailed [51]. Nanostructured (4–10 nm) anatase powders were obtained having a N content up to 1.5 at. % and W up to ca. 22 at. and tested in the partial oxidation of toluene and styrene. Analysis of the effect of calcination temperature in the 400–600 °C range provided evidence of the activity maximization at the lower calcination treatment, irrespective of the illumination wavelength [52]. A N/W 0.1/10 at. % content of the anatase phase optimized activity. This was ascribed to a combined effect of the decrease of the band gap energy and the concomitant increase of radical hydroxyl-type species (in turn derived from a decrease of charge recombination).

**Figure 3:**
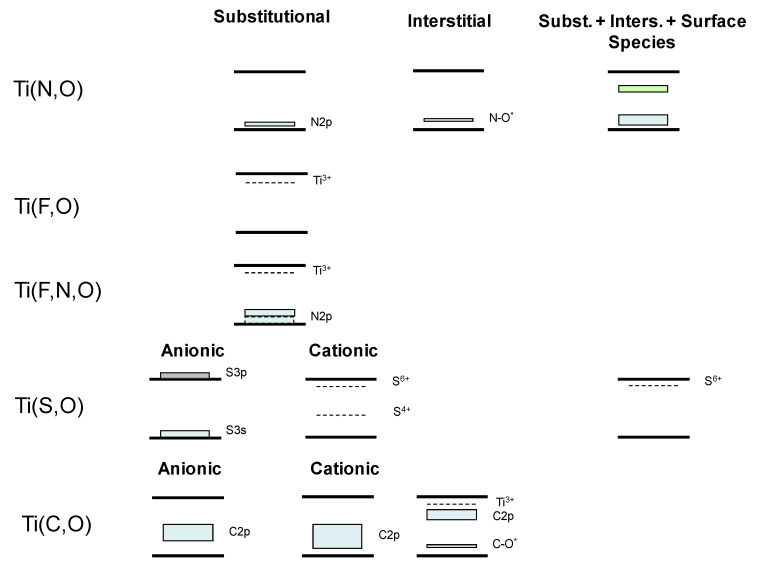
Schematic representation of the electronic effects generated by non-metal doping in anatase-based systems.

The cases of halides [53,54,55,56] is relatively simple. Fluoride incorporation at the bulk of anatase generates Ti^3+^ species on the gap for charge compensation (Figure 2). On the other hand, F presence at the surface has a strong effect in the primary particle shape of anatase, favoring the exposure of the (001) plane [57,58,59]. S doping has also been explored and, contrarily, is a structurally complex system. To a first approximation, Sulphur doping parallels the complexity of N-containing one in terms of the chemical species potentially present as well as electronic effects. S doping usually considers anionic (S^2−^; S_2_^2−^) species [60] and cationic (S^4+^; S^6+^) substitutional/interstitial-type [61] species, in addition to, at least, sulfate surface species. S stabilizes the anatase polymorph (retarding rutile appearance upon calcination), increases light absorption and, in both anionic and cationic positions, reduces charge carrier recombination. Figure 3 summarizes the electronic situation for the mentioned cases. Sulphur combination with Fe has been shown to be particularly effective for UV and visible light utilization in photo-catalysis [62].

C is another complex case, experimental approaches indicate the presence of surface carbonate and carbonaceous species, as well as lattice-bound carbon species [63,64]. Lattice positions can be anionic and cationic substitutional positions as well as interstitial ones. Their electronic effects are detailed in Figure 3. Apparently, most efficient photocatalysis is achieved in presence of localized states near the valence band. This similarity with nitrogen doping is not only a characteristic of TiO_2_ based catalysts but it is apparently the case for ZnO [65]. Co-doping of C and Fe has been also essayed to obtain single phase nanostructured (primary particle size of 12–16 nm) anatase powders [66]. C atoms were detected at substitutional and interstitial positions of the anatase while Fe occupied Ti cation lattice positions. Materials were tested for malachite green photo-degradation. Authors observed maximum activity using a catalyst having a doping content of ca. 8/2 C/Fe wt.%. In these co-doped samples, carbon is responsible for appearance of localized states near the conduction band while Fe has multiple roles, particularly affecting capture electrons and decrease recombination of charge carriers. The cooperation of the two alien atoms opens a pathway for visible light utilization and to optimize the charge carrier fraction utilized to oxidize the target molecule. The co-doping of zinc oxide is also frequently investigated, and the C and Fe pair is a case under investigation. An Fe content close to 2.1 wt.% leads to maximum activity for 2,4,6-trichlorophenol elimination under visible light illumination. For this formulation, carbon and Fe ions trigger the appearance of localized levels near valence and conduction bands, respectively, facilitating visible light absorption and improving activity with respect to single-doped and bare oxide reference materials [67].

## 3. Composite Photocatalysts

Composite photocatalysts can have a significant number of different families. A first one considers the coupling of two (or several) semiconductors. Using titania as the base component we can distinguish between systems oriented to couple titania with visible or infrared oriented components. A second family can couple titania with metals and is particularly oriented to use the surface plasmon resonance to enhance simultaneously UV and visible (sometimes near IR) capabilities of the photocatalyst. A third family utilizes polymers as the base component of a composite system. In all cases, the interface among components plays a critical role in favoring activity [68].

Considering the use of UV and visible light, main examples of the first family of TiO_2_-based samples present a positive photocatalytic response (higher activity than the bare titania reference). Such an effect has been achieved by a well-controlled interaction of titania with minor oxide entities such as SiO_2_ [69,70], CuO_x_ [71,72], NiO [73], ZnO [71,74], Bi_2_O_3_ [75,76,77], CeO_x_ [77,78,79], or WO_3_ [80]. FeO_x_-containing semiconductors are also actively investigated however the behavior of such systems combine frequently photocatalysis with photo-Fenton [81]. More complex formulations consider the combination of titania with, for example, heteropolyacids [82,83], vanadates such as BiVO_4_ [84,85] or complex oxides such as CuNiOx [86] and FeAlO_3_ [87]. Similar catalysts have been studied for ZnO-based materials. More specific are those having two Zn-based semiconductors such as ZnWO_4_ [88] or ZnFe_2_O_4_ [89] combinations with ZnO. In these cases, to a first rough approximation, the UV activity is controlled by titania (or ZnO) and can be improved if charge separation takes place between the two components after light absorption. This requires specific arrangements of the conduction and valence bands of the components, being among the most successful those of type-II (which separates physically electron and holes created in both components) or Z-type schemes (which facilitates the selective annihilation taking place between electrons of one component and holes of the other). The exact contact between titania and the above-mentioned second components is strongly size-dependent, and current information is relatively limited [1,17]. Visible activity is, on the other hand, usually achieved by the light excitation of the second component although the different (local) structure of titania and the interface between components can play an active catalytic role.

**Figure 4:**
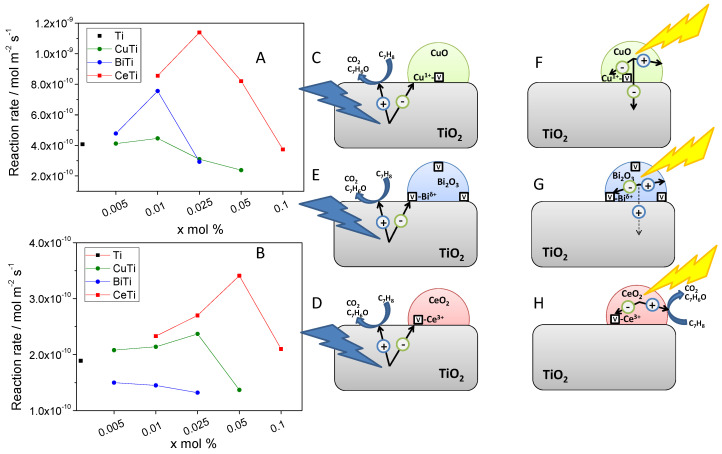
Reaction rate for of the samples (CuO-, Bi_2_O_3_- and CeO_2_-TiO_2_) and TiO_2_ reference prepared by microemulsion method under UV (**A**) and Sunlight-type (**B**) illumination conditions. Schematic representation of the interface role in the composite samples under UV (**C**–**E**) and Visible (**F**–**H**) irradiation. Reprinted with permission from [73]: Muñoz-Batista, M.J.; et al. *ACS Appl. Mater. Interfaces.* **2016**, *8*, 13934–13945. Copyright © 2020 American Chemical Society.

As an illustrative example of the situation we highlight a study using three of the aforementioned oxides (CuO, CeO_2_ and Bi_2_O_3_) present on the surface of an active (and dominant component on percentage) anatase TiO_2_ component [77]. Under UV, activity was optimized at somewhat low concentrations (x mol% below 2.5 mol%; Figure 4A). Under sunlight-type irradiation, the maximization of activity required catalysts with higher concentrations of the surface oxides (2.5 mol% for CuO and 5 mol% for CeO_2_; Figure 4B). Although an enhancement of the absorption of visible light photons was confirmed using optical spectroscopies, the Bi_2_O_3_-TiO_2_ system did not improve (for all contents) the activity of the pure TiO_2_ reference. Figure 4C–H depicted a schematic photo-handling scheme taking place for CuO-, Bi_2_O_3_- and CeO_2_-TiO_2_ composite samples under illumination. Under UV illumination conditions (Figure 4A–E), the active role of the interface for the efficient capture of electron leads to an increase of the concentration of holes and hydroxyl radicals in the surface of the anatase and finally triggers the activity enhancement. Under sunlight-type illumination, a more complex situation appears to occur (Figure 4F–H). The structural and electronic characterization of the samples provided evidence that the CeO_2_-TiO_2_ interface has a positive role in charge handling. Contrarily, such electronic interaction between components negatively affected the chemical behavior of the Bi_2_O_3_-TiO_2_ samples. For CuO-TiO_2_, negative effects on activity could come from the annihilation of charge and concomitant decrease of available holes at the surface of the major TiO_2_ component. As illustrated in Figure 4, these numerous charge-related phenomena taking place under the simultaneous excitation by UV and visible wavelengths inform that activity relies on the physico-chemical properties of the components, and, critically, on the interaction between them.

**Figure 5:**
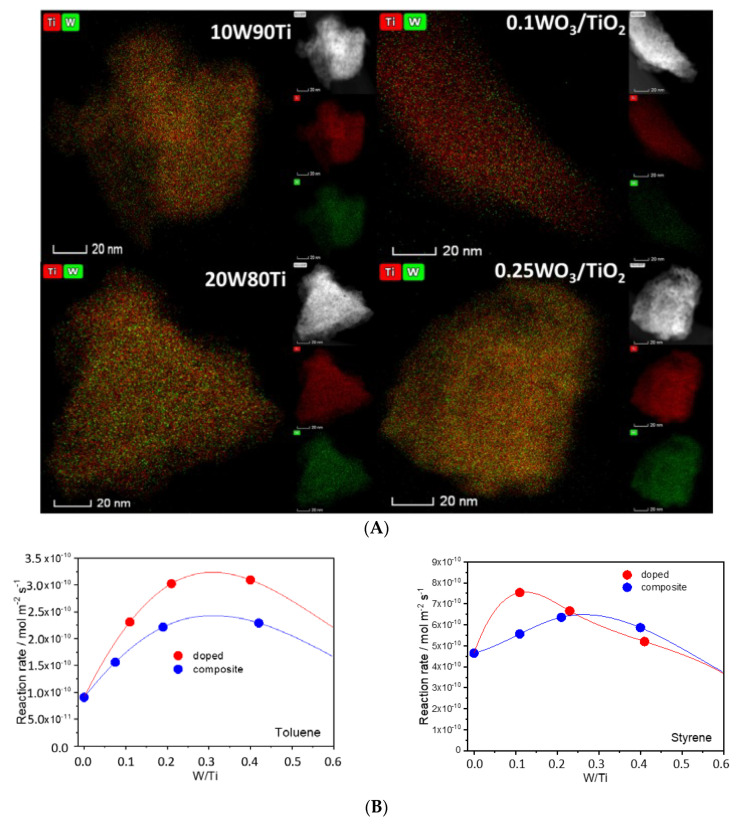
(**A**) EDS-STEM images for W-Ti materials. 10W90Ti and 20W80Ti label doped samples with W/Ti atomic ratio of 0.11, 0.23, respectively. 0.1WO_3_/TiO_2_ and 0.25WO_3_/TiO_2_ are composite catalysts with 0.11, 0.24 WO_3_/TiO_2_ molar ratio, respectively. A dark field STEM image is included at the right-hand top part of each map. (**B**) toluene and styrene photo-catalytic elimination reaction rate as a function of the W/Ti atomic ratio of the samples. Reprinted with permission from [86]: Caudillo-Flores, U.; et al. *Appl. Catal. B: Environ.* **2019**, *245*, 49–61. Copyright © 2020 Elsevier.

A singular contribution makes a careful comparison between doped and composite W-containing TiO_2_ anatase catalysts [90]. This would attempt to compare the difference of surface tungsten species obtained in a pure doped system with those deposited onto the titania oxide. Figure 5 summarizes information rendered by electron diffraction for doped (W-TiO_2_) and composite samples (WO_3_/TiO_2_). The physico-chemical analysis of the catalysts probes the differences in the W-containing entities in these two types of samples. The dark field microscopy images included in Figure 5 identified the specific geometrical/structural location of W in doped and composite samples, showing the structural differences of having isolated atoms located at titania surface positions (doped samples) from those where always W-O-W (clustering) is obtained (composite samples). The activity of the doped/composite samples having equal W/Ti ratio is also presented in Figure 5. Strong differences are observed between the two types of samples, a fact that emerges from the different handling of light and surface properties [90].

**Figure 6:**
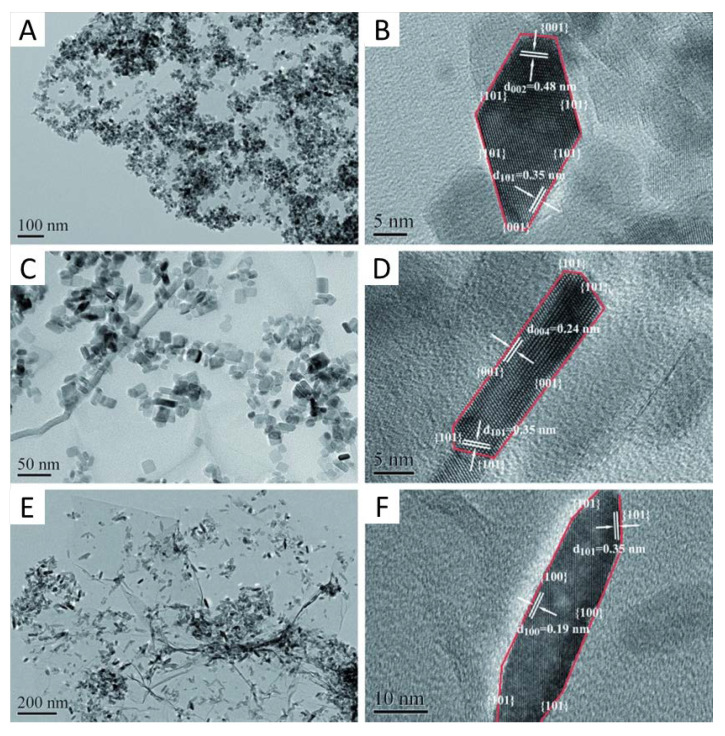
TEM and HRTEM images of: (**A**,**B**) TiO_2_-101-G, (**C**,**D**) TiO_2_-001-G, and (**E**,**F**) TiO_2_-100-G. Reprinted with permission from [97]: Liu, L.; et al. *ChemSusChem* **2014**, *7*, 618–626. Copyright © 2020 Wiley.

Combination of titania with carbon containing semiconductors such as carbon nitride [91,92,93,94,95] and graphene [96,97,98,99] or graphyne-type materials [100,101,102] can also be highlighted in the context of the UV-visible combination. Many oxides, for example, CeO_2_ were also tested in combination with carbon layers [103] or carbon nitride [104]. Considering pure anatase, the interaction with carbon nitride has been analyzed but conflicting results are present in the literature. As reviewed in ref. [4], both type II (with electrons transferred to titania and holes to carbon nitride) and Z-scheme (with electrons from titania and holes from carbon nitrate suffering annihilation) junction appears to operate under UV illumination. No obvious physico-chemical grounds have been presented in order to justify such difference. Under visible illumination it appears that charge separation takes place with electrons going from carbon nitride to titania. Operation under sunlight improves the parent systems and this has been particularly ascribed to the electronic contact between the materials, as well as other positive effects of carbon nitride incorporation into the composite and related to morphological and adsorption properties [91,92,93,94,95]. In the case of graphene-titania, similar positive effects have been described to consider the higher activity displayed with respect to the parent reference systems [96,97,98]. An interesting study analyzed the specific interaction of shape-controlled anatase particles exposing specific surfaces to graphene [105]. As shown in Figure 6, the control of the shape of the anatase nanoparticles triggers the contact of the anatase with the carbon nitride support using specific surfaces planes, (101), (001) and (100). Activity is strongly promoted by the presence of graphene. Activity is shown to follow the order (100) > (101) > (001), which is not the activity order for pure anatase samples. An improved charge transfer for the case of the (100) surface due to the generation of close Ti-C carbon contacts is claimed to be the main reason for the high activity of the composite system. Again, this proves that the contact between phases is strongly dependent on local order details, which are not easily predicted using current knowledge. The detailed information at nanometric level may permit us to understand the effect of the interface on charge handling and would thus allow us to rationalize the charge behavior of the composites of titania and carbon-containing materials.

**Figure 7:**
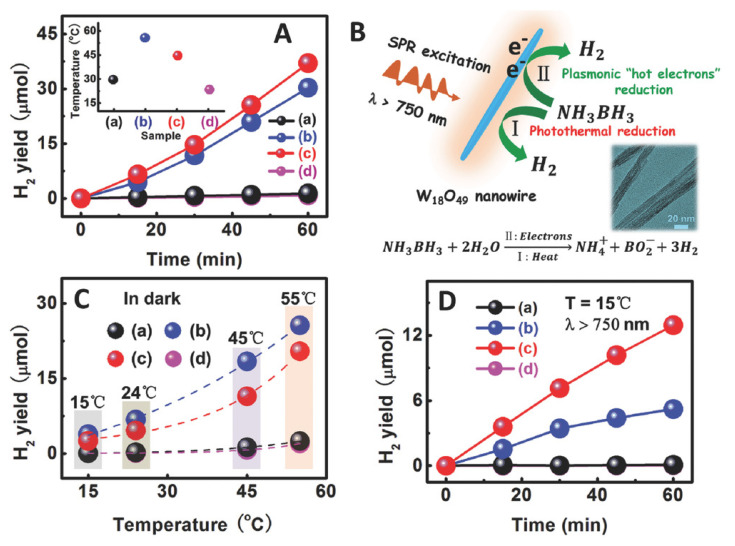
(**A**) Time-dependent H_2_ generation from NH_3_BH_3_ aqueous solution over different samples upon IR-light irradiation (λ > 750 nm) (the inset shows the corresponding catalytic reaction temperatures); (**B**) schematic of the catalytic mechanism for H_2_ generation from NH_3_BH_3_ molecules over the plasmonic W_18_O_49_ NWs (the inset shows a TEM image of the W_18_O_49_ NWs); (**C**) catalytic H_2_ generation from NH_3_BH_3_ aqueous solution at different temperatures for 1 h over different samples without light irradiation; (**D**) time-dependent H_2_ generation from NH_3_BH_3_ aqueous solution at 15 °C over different samples upon IR-light irradiation: (a) TiO_2_ NFs; (b) W_18_O_49_ NWs; (c) W_18_O_49_/TiO_2_ branched heterostructure; (d) without a catalyst. Reprinted with permission from [102]: Zhang, Z.; et al. *Adv. Mater.* **2018**, *30*, 1705221. Copyright © 2020 Wiley.

Additional composite systems relay in the combination of the UV-oriented TiO_2_ and infrared absorbing semiconductors. Sulfides such as MoS_2_ or In_2_S_3_ have been used in this context [106,107]. Regarding oxides, we can highlight the cases of Ag_2_O [108], and WO_x_ [109,110]. These systems and particularly the latter are real, full-spectrum catalysts, with utilization of the UV-visible-nearIR electromagnetic range. In addition, WO_x_-TiO_2_ has been tested in ternary configurations with graphene oxide in order to enhance activity upon all illumination conditions [111]. Defective W(VI) oxides are known to present a strong absorption capability along visible and infrared regions, and are also photocatalytically active. In Figure 7 it is shown that the W-Ti composite excited under near infrared (750 nm) light combines thermal and light-related effects. Thermal effects are not as strong as in the pure WO_x_ reference but they clearly contribute to photocatalytic activity. This appears as a mixture of thermal and light effects due to the non-radiative and radiative de-excitation taking place under visible and IR illumination conditions. The effect is not a simple sum and a clear synergy comes out from the analysis of Figure 7 [110]. Such a behavior was also present in Ru-RuO_x_ core-shell structures supported in anatase TiO_2_ [112]. The heat-light synergy is a complex phenomenon which is observed the presence of plasmonic type components but also adequate handling of light-heat transformation through the phonons of the solids as well as surface chemical species. Additionally, non-plasmonic materials could also provide the basis of thermo-photo catalysts using infrared light as heat and/or light primary sources. Ceria-based materials constitute a paradigmatic example, with thermo-photo activity mostly related to defect chemistry, although the details are essentially unknown [113,114,115,116].

**Figure 8:**
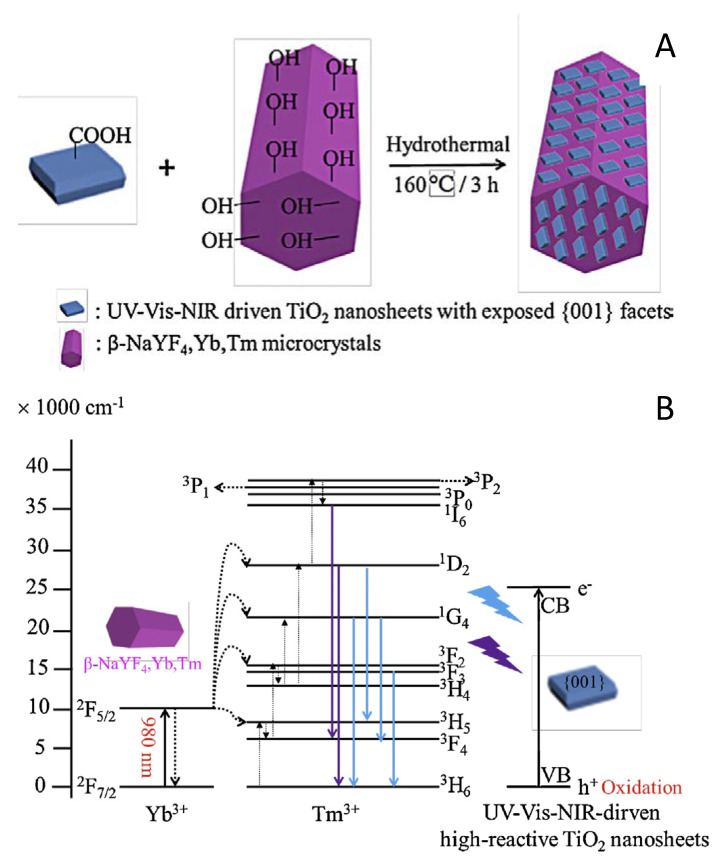
Schematic illustration of preparation method and structure of NaYF_4_@TiO_2_ photocatalyst (**A**). Schematic illustration of energy transfer mechanism from Yb^3+^-Tm^3+^ doped hexagonal phase NaYF_4_ microrods to {0 0 1} facets dominated UV–vis–NIR driven TiO_2_ nanosheets under the 980 nm light irradiation (**B**). Reprinted with permission from [109]: Wang, W.; et al. *Appl. Catal. B* **2014**, *144*, 379–385. Copyright © 2020 Elsevier.

Other composite systems relay in the used of solid up-converters. An illustrative example concerns the NaYF4:Yb^3+^, Tm^3+^@TiO_2_ core-shell structured photocatalyst. The characterization results showed that the core–shell structured composite consisted on (hexagonal phase) NaYF_4_:Yb^3+^, Tm^3+^ microrods and anatase TiO_2_ nanosheets with exposed high-reactive {001} facets (Figure 8). The new photocatalyst gives higher photocatalytic activity than the (components) physical mixture and pure TiO_2_ for phenol and RhB elimination under NIR and (to a lesser extent) simulated sunlight irradiation. The authors proposed a mechanism to explain the enhancement in the photoactivity reached with the NaYF_4_:Yb^3+^, Tm^3+^@TiO_2_ photocatalyst, as summarized in the schematic illustration presented in Figure 8. In this figure, the up-converter supplies UV and visible (blue) photons to the titania counterpart [117]. Other relatively similar works are rhombic hierarchical YF_3_/TiO_2_ [118], β-NaYF_4_:Yb^3+^,Tm^3+^/Er^3+^-TiO_2_ [119], or NaYF_4_:Yb^+3^,Nd^+3^ @ TiO_2_ [120] composite systems.

On the other hand, the up-conversion core catalysts have not only been combined with TiO_2_ but also with other materials to obtain hybrid or composite ternary systems. Up-conversion/C-TiO_2_ ternary composite systems have been studied in detail. In a first work [121], a (Yb^3+^,Er^3+^)-NaYF_4_/C-TiO_2_ composite system photoactivity was evaluated for NOx destruction reaction under UV, visible and near-infrared light illumination conditions. The results showed that when the C-TiO_2_ photocatalyst was combined with the (Yb^3+^,Er^3+^)-NaYF_4_ up-conversion phosphor, the visible absorption of C-TiO_2_ was significantly improved up to 900 nm, presenting a nice correlation with the photoluminescence spectrum of (Yb,Er)-NaYF_4_ excited with 980 nm. For this reason, the (Yb^3+^,Er^3+^)-NaYF_4_/C-TiO_2_ composite presented excellent deNOx ability not only under the irradiation of visible and UV lights but also NIR light, being much superior to those of pure C-TiO_2_, P25 and even the (Yb^3+^,Er^3+^)-NaYF_4_/N-TiO_2_ composite. In the second work [122], the authors mixed the blue color (Yb^3+^,Tm^3+^)-NaYF_4_ (named B-UP), green color (Yb^3+^,Er^3+^)-NaYF_4_ (named GUP) and red color (Yb^3+^,Er^3+^)-Y_2_O_3_/YOF (named R-UP) up-conversion phosphors with C-TiO_2_ using a weight ratio of 1:1 The photocatalytic activities of the composites were evaluated in the degradation of RhB and the elimination of NO gas under NIR light irradiation. According to photocatalytic results, the photonic efficiency under infrared light was much higher than the corresponding observables obtained under UV and visible light. The green light emitting up-conversion phosphor-C-TiO_2_ composite presented superior photocatalytic performance over blue and red ones.

Another family of materials concerns the anatase-based composite systems with a second plasmonic-type component. Most typically plasmonic components consist of metals; we previously mentioned those based in semiconductors, such as tungsten-based ones. A plasmonic-based catalytic system based in metals is mostly used to profit from UV and visible light, although here we will show examples that can also profit from near infrared wavelengths. Under UV illumination, titania and most typical semiconductors can improve charge separation through the electron storage capacity of the metal and the subsequent beneficial effects connected with the higher lifetime of hole-related species located at the semiconductor. Under visible illumination, the metal is the component able to absorb light and electrons can be transferred to the semiconductor phase [1,6]. Of course, metals are also frequently utilized in multi-component catalysts (and not only with a main UV- absorber photocatalyst) in order to efficiently harvest the full solar light spectrum. The most utilized (plasmonic or not) metals as photo-catalytic co-catalysts are Au, Ag, Pt, Pd, or Cu as well as their binary combinations [1,5,123,124].

Considering metals gold is a classical plasmonic-type choice. Asymmetric Janus nanostructures containing a gold nanocage (NC) and a carbon–titanium oxide hybrid nanocrystal (AuNC/(C–TiO_2_)) exhibited 3.2 times higher photoactivity than that obtained with the C–TiO_2_ in the photocatalytic aerobic oxidation of isopropanol under visible light. Electromagnetic field simulations and EPR results indicate that plasmon–photon coupling is largely amplified at the interface between the AuNC and C–TiO_2_ components, leading to the enhanced generation of energetic hot electrons utilized in the photocatalytic reaction [125]. Nanoparticles of gold over titania also showed exceptional activity in bio-alcohol reforming under UV and solar light conditions, optimizing activity for sizes near 5–6 nm [126]. An interesting ternary system using this metal is the NaYF_4_(Yb^3+^/Er^3+^)-TiO_2_-Au [127,128]. In one work, a series of Au decorated NYF@TiO_2_ core@porous-shell microspheres (0.5, 1, 2, 3 wt.% Au) were presented. The photocatalytic efficiency of these new nanohybrid catalysts toward methyl orange (MO) degradation was examined, and the significant enhancement was observed with respect to the benchmark photocatalyst P25, as well as NYF and NYF@TiO_2_ samples. The highest MO degradation was obtained with the material with 1 wt.% of Au under NIR and solar irradiation. The enhanced activity was attributed to synergistic effects from nanocomponents arranged into the nanostructured architecture in such a way that favors the efficient charge/energy transfer among nanocomponents and largely reduced charge recombination [127]. Recently, the (NaYF_4_:Yb^3+^/Er^3+^)-Au/TiO_2_ nanotube-type material was synthesized and its photocatalytic activity compared with the TiO_2_ NTs (nanotubes) and Au/TiO_2_ for the RhB degradation under NIR illumination. The results indicated that the up-converter-Au/TiO_2_ NTs sample exhibited outstanding photocatalytic activity in the degradation of RhB but no obvious degradation was observed in the presence of TiO_2_ NTs and Au/TiO_2_ NTs. These results indicate that NIR light can be exclusively utilized by (NaYF_4_:Yb^3+^/Er^3+^)-Au/TiO_2_ NTs [128].

Silver is another plasmonic metal with frequent utilization in the field of photocatalysis. A series of Ag-TiO_2_ photocatalysts were evaluated for phenol degradation under visible irradiation. Samples always contained a combination of Ag metallic and Ag^+^ chemical states. Optimum activity was reached using a silver loading of 6.5 mol% (measured as AgNO_3_ used during the preparation stage). Such optimum was correlated with the maximization of the amount of silver in Ag^0^ form. More specifically, the authors suggested that the activity improvement connects with the presence of silver metal (due to enhancing the electron–hole separation and surface plasmon resonance as well as the presence of surface carbon working as a light sensitizer) [129]. Zerovalent metallic silver nanoparticles with hemispherical shape were observed in a series of g-C_3_N_4_/AgX (X = 2, 5, 10 and 12 wt.% of Ag) materials. The best photoactivity was obtained with the sample with 10% of Ag, improving the performance of the bare carbon nitride by ca. 2-fold for degradation of dyes. In a subsequent work, the g-C_3_N_4_/Ag10% material was the base for a ternary catalyst with MoS_2_ quantum dots. An enhanced photocatalytic activity was observed for the hybrid photocatalyst. This was ascribed to the charge handling Z-scheme taking place in the system, with Ag nanoparticles acting as the charge separation center [130]. A ternary system containing silver has been extensively studied. A different ternary system is a NaYF_4_:Yb^3+^, Tm^3+^@TiO_2_/Ag core@comby shell nanostructure. The R6G dye degradation results demonstrated that the composite exhibits excellent photocatalytic activity as the up-converter core can efficiently converts NIR light into UV light [131]. NIR and UV-visible light induced photocatalytic activity was observed for a NaYF_4_:Yb^3+^,Tm^3+^@TiO_2_/Ag composite. The nanocomposite showed photocatalytic activity for Rhodamine B degradation under full solar irradiation. Corresponding activity measurement appeared to increase UV and visible light activity with respect to photocatalyst(s) based on Ag-titania. The enhancement was interpreted with the help of photoluminescence and ascribed to the NIR light influence in the overall photocatalytic reaction taking place in the NaGdF_4_:Yb^3+^: Er^3+^/Ag/TiO_2_ composite [132].

Closely related to the above described silver-containing materials are Ag/AgX/semiconductor ternary materials. The silver coupling with a silver halide has been subjected to many investigations. As an example, an efficient and stable visible-light-driven Ag/AgX/graphene oxide, GO, (X = Br, Cl) plasmonic photocatalyst was assembled. The XPS results confirmed the existence of metallic Ag^0^ in the Ag/AgX/GO hybrid nanocomposites with a surface molar ratio Ag^0^ to Ag^+^ of 1:16. Enhanced photocatalytic performance for the photodegradation of MO dye under visible-light illumination was shown by these materials with respect to Ag/AgX references. This could be attributed to the high adsorptive capacity of Ag/AgX/GO, the smaller size of Ag/AgX “nanoparticle” in the hybrid nanocomposites, the reinforced charge transfer, and the suppressed recombination of electron/hole pairs caused by the Ag^0^ presence in Ag/AgX/GO [133]. The Ag/AgX couple was also supported on other semiconductors. Presence of 10–20 nm Ag^0^ within Ag@AgCl quantum dots (QDs) were observed to be evenly dispersed on the surface of Bi_2_WO_6_. The Ag@AgCl (20 wt.%)/Bi_2_WO_6_ sample exhibited the best photocatalytic activity, degrading 97.6% of rhodamine B (RhB) after irradiation for 2 h, which was 1.33 times and 1.32 times higher than that of Ag@AgCl and Bi_2_WO_6_ photocatalysts, respectively. This was attributed to the excellent photocatalytic activity due to the synergetic effect of Bi_2_WO_6_, AgCl, and Ag nanoparticles [134]. Ag/AgBr/TiO_2_ composite catalysts were tested for elimination of liquid phase aqueous phenol and gas-phase acetaldehyde. The composite showed an efficient photo-generated charge transfer between AgBr and TiO_2_ under both UV–visible or visible illumination conditions. This in turn facilitated charge separation and the enhancement of the activity [135]. Finally, Ag/AgX/SrTiO_3_ (X = Cl, Br) plasmonic photocatalysts were prepared by microemulsion and metallic Ag generated after a subsequent irradiation step. The degradation of MO and RhB dyes was essayed under visible light illumination, with the system containing AgBr showing higher activity. The enhanced activity was ascribed to the active role played by the AgBr component working together with the surface plasmon resonance of the Ag nanoparticles [136].

In addition, Pt has attracted attention in terms of using metal as a second component in composite catalysts. Platinum does not present (at least for nanosized range nanoparticles) a pure plasmonic optical behavior like the above-mentioned metals, but it is broadly used in the context of utilizing UV and visible light. Metallic Pt nanoparticles (3–5 nm) deposited on the surface of a commercial TiO_2_ (Fluka) showed good activity for 2-propanol photo-degradation. Presence of the metal increased the activity ca. 1.6 times with respect to bare oxide. The Pt role was twofold, firstly it provides adsorption sites for 2-propanol and secondly, it acts as electron sink to facilitate separation of charge carrier species [137]. In other work, Pt nanoparticles supported over a N-doped TiO_2_ were investigated, particularly the effects of the Pt loading content and pH of the (methanol/water) reaction medium on the performances of the photocatalysts for hydrogen evolution. The results showed that the addition of Pt onto the TiO_2−x_N_x_ surface (optimum loading of 0.2 wt.%) can increase the photocatalytic reaction and enhance hydrogen evolution. The photocatalytic reaction appears to be favored in the neutral pH range [138]. A rather nice contribution analyzed the catalytic effect of several variables such as the amount of Pt precursor, TiO_2_ properties, deposition method (reverse microemulsion and wet incipient impregnation) and reducing agent on size, distribution, and chemical state of deposited Pt nanoparticles. XPS results confirmed that samples prepared by microemulsion showed much higher binding energy values for platinum than the other preparation method. Degradation of phenol under UV-visible illumination was shown to depend on Pt particle size. Optimum particle size and maximization of activity was achieved using the microemulsion method [139]. Interestingly, Pt can be deposited over anatase TiO_2_ selectively over specific surface planes (like (101)), favoring specific reactions like hydrogen production [140]. Pd promotion of TiO_2_ activity was also investigated [141]. The work analyzed the control of the metal particle size and showed that an average particle size of 2.4 nm improves activity significantly with respect to bare TiO_2_, particularly under visible light [141].

**Figure 9:**
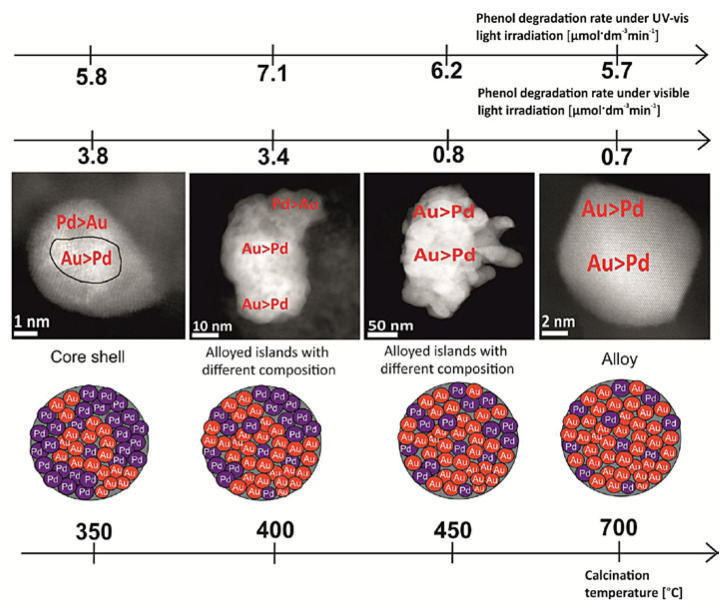
The effect of calcination temperature on the structure of Au/Pd bimetallic nanoparticles and photoactivity of Au/Pd-TiO_2_ nanocomposites. Reprinted with permission from [135]: Cybula, A.; et al. *Appl. Catal. B: Environ.* **2014**, *152–153*, 202–211. Copyright © 2020 Elsevier.

Bimetallic particles have also been utilized in composite materials in order to add visible and near infrared capabilities to the typical UV of the main semiconductors such as titania. Gold is present in a significant number of contributions. Bimetallic Ag/Au modified-titanium oxide photocatalysts improved the phenol photo-decomposition performance of monometallic references, obtaining the highest reaction rates with the samples Ag/Au-TiO_2__4 and Ag/Au-TiO_2__5 which contain different Au/Ag ratios (1.5/0.5 and 1.5/4.5 respectively) supported over the so-called “TiO_2__4” (mixture of rutile and anatase) and “TiO_2__5” (rutile) oxide solids, respectively [142]. Bimetallic (Au/Pt) nanoparticles were also prepared on titania using a microemulsion procedure. Optimum phenol degradation was achieved with the sample calcined at 450 °C with an Au/Pt ratio of 0.5/0.1 mol%. Size-controlled Au/Pt nanoparticles together with a significant amount of electronegative gold species (Au^δ−^) resulted in higher photoactivity. Also, the samples prepared using the above supports overperform others Au/Pt-TiO_2_ composites with the same Au/Pt ratio over different TiO_2_ supports (P-25, ST-01, TiO-5, TiO_2_ nano-tubes) [143]. In a somewhat parallel contribution, Au/Pd nanoparticles supported on TiO_2_ were investigated paying attention to the calcination temperature. Increasing it from 350 to 700 °C deteriorates activity. A modest decrease under UV and quite significant decrease under visible light for phenol photo-degradation can be seen in Figure 9. Catalysts calcined at 350 and 400 °C showed the highest photocatalytic activity for phenol degradation under visible and UV light, respectively. An in-situ EPR study examined the formation of radical species under illumination. This tool found that the O^2−^ radical species is mainly responsible of the pollutant degradation under all illumination conditions tested. The study was completed with the help of HAADF-STEM (High-angle annular dark-field scanning transmission electron microscopy) and TEM microscopies. Results pointed out that the Pd to Au ratio in the surface layer of Au/Pd nanoparticles decreased from 5:1 to 1:4 with calcination temperature going from 350 to 700 °C, respectively. The overall analysis indicates that increasing calcination temperature caused segregation of metals and gold-enrichment in the outermost region of Au/Pd bimetallic nanoparticles. Optimum photoactivity is connected with the adequate degree of mixing of both metals and the dominant presence of Pd at surface layers (Figure 9) [144].

Recently, novel Ag-Cu bimetallic alloy NPs decorated β-NaYF_4_: Yb^3+^, Tm^3+^ (NYFT) @TiO_2_ micro-rods were prepared by a surfactant-assistant sol–gel method and H_2_ reduction process. Under near-infrared (NIR) light irradiation, Ag-Cu alloy supported NYFT exhibited significantly enhanced photocatalytic activity in comparison with other samples (NYFT, NYFT@Ag and NYFT@Cu), and water disinfection involving 100% inactivation of bacteria was achieved within 8 h. The increase in the photoactivity was assigned to the synergistic effect taking place between the up-conversion material and alloy NPs. In the NYFT@Ag-Cu composite, NYFT could convert NIR into UV and visible light. TiO_2_ absorbed UV emissions and produced electrons and holes. Electrons in the CB of TiO_2_ could first transfer to Ag then to Cu, which would effectively inhibit recombination of charge carriers and increase their lifetimes [145]. Bimetallic co-catalysts presenting Pt as a major component find application in numerous photocatalytic processes. As an example, Pt-Pd [146] and Pt-Ru [147] co-catalysts supported on nonmetric (ca. 10 nm) anatase were tested in the photoproduction of hydrogen from methanol: water mixtures. For Pt-Pd, optimum activity can be ascribed with the formation of binary alloys having disorder fcc type structure. For Pt-Ru, Pt in a metallic state was located in the proximity of Ru oxide species. Irrespective of the structural situation, the binary systems improve charge separation and overperform the corresponding monometallic system under both UV and sunlight operating conditions. We can also mention a Fe/La/Zn@GO trimetallic nanocomposite, active in the elimination of the phenylhydrazine organic pollutant under sunlight illumination [148].

To end this section, we will briefly mention photo-catalytic oxide systems introduced in polymer matrixes. Titania is the main semiconductor used in this case. The incorporation of nanostructured titanium oxide has been utilized in several polymers such as ethylene vinyl alcohol (EVOH) [149], polypropylene (PP) [150], polycaprolactone (PCL) [151] or polyvinylidene difluoride (PVDF) [152] to generate self-cleaning and self-degrading materials. The analysis of the biocidal properties showed that a good contact between titania can make the whole surface of the composite material biocidal and this provides the grounds for making active surfaces with wide application and at the same time allowing a controlled degradation of the polymer in order to avoid environmental problems [153]. Another important field of application relates to water treatment using membrane materials. Ultrafiltration membranes using such composite materials are the most commonly reported. Using titania [154,155] or ZnO [156] UV active materials were presented while doping with Nd or graphene oxide (GO) of titania were utilized to trigger the membrane functional properties upon visible light illumination [157]. Finally, other functional composite materials can be exemplified by the ZnO/SiO_2_ composite introduced into an ETHOCEL resin as UV light blocker [158].

## 4. Conclusions and Outlook into the Future

In this contribution, we survey the work carried out in the photocatalysis field and oriented it to the use of sunlight as the energy source of the photo-chemical process. In this immense field of research, we focus on titania-based materials as the most promising candidate to achieve this goal. Nevertheless, titania is a UV absorber which can be the base of a universal highly efficient system but requires significant engineering to achieve a fruitful use of the full spectrum of the solar light. As the main tools to achieve this goal, we reviewed doped and composite materials as well as the combination between these two approaches.

Doping of the main semiconductors such as titania has been extensively studied. Cation and/or anion doping related to the insertion of metal and non-metal ions in substitutional/interstitial surface and/or bulk positions was used to modify the band gap energy of titania and to create efficient localized electronic states to achieve visible light absorption and activity. A specific case corresponds to up-conversion cations, which allows the utilization of near infrared wavelengths. Similarly, composite materials were utilized to achieve fruitful use of visible and near infrared regions of the solar spectrum. Composite systems using semiconductors with low band gap, or displaying plasmonic or up-conversion capabilities, are highlighted as rather powerful systems. Metal-titania systems were also analyzed in terms of their potential for visible and/or infrared light (in addition to the UV). Ternary systems combining metals, low band gap or up-converters semiconductors and (doped or not) titania are mentioned as potentially the most useful full spectrum photo-catalysts. They require complex preparation procedures and fine tune of the contact between components but would allow future progress of the field. Finally, we briefly reviewed the use of polymers as matrixes to embedded photo-catalytic oxides and generate functional materials with improved capabilities.

Although the full profit of the solar light is an extraordinary challenge, the progress in the field has reached an extraordinary level, mostly relaying in the utilization of the above-mentioned complex materials. The combination of all relevant technologies considering the management of the electronic properties of titania (mainly although not exclusively band gap energy) jointly with the combination of this base material (as well as other base materials such as zinc oxide) within composite systems having (the above-described) specific components to profit from visible and infrared photons seems a clear route to achieve a good photocatalyst that is able to utilize efficiently photons in the complete electromagnetic range of sunlight. Nevertheless, optimization of activity requires the fine tuning of the light-matter interaction and chemical properties of such complex, final materials. This task can be achieved by a careful design and engineering of the structural/electronic properties of each component, the corresponding interfaces among components as well as of the (most) active surfaces to be exposed to the reactive atmosphere. New synthesis methodologies and the advancement in the physico-chemical characterization of the photocatalytic materials would guide out work in achieving this goal.
